# The gut virome and regulatory T cell axis in health and systemic disease

**DOI:** 10.20517/mrr.2026.20

**Published:** 2026-06-22

**Authors:** Dandan Bi, Shaochen Yu, Mengjie Zhang, Yuting Huang, Ziyue Dou, Beibei Tian, Jian Lu

**Affiliations:** ^1^Department of Internal Medicine, Beijing Jishuitan Hospital Guizhou Hospital, Guiyang 550014, Guizhou, China.; ^2^Department of Emergency and Critical Care Medicine, Chuzhou Integrated Traditional Chinese and Western Medicine Hospital, Chuzhou 239000, Anhui, China.; ^3^Department of Gastroenterology, The First Affiliated Hospital of Anhui Medical University, Hefei 230022, Anhui, China.; ^#^These authors contributed equally to this work.

**Keywords:** Gut virome, bacteriophages, host immunity, regulatory T cells, intestinal inflammation, immune regulation, microbiome

## Abstract

The gut virome, comprising bacteriophages and eukaryotic viruses, represents a complex and dynamic component of the intestinal microbiome whose functional significance has long been underestimated. Emerging evidence highlights the gut virome as a pivotal modulator of the host immune system, particularly in regulating the balance and function of regulatory T cells (Tregs), which are essential for maintaining immune homeostasis. This review distinguishes two mechanistic axes by which the virome influences Tregs: (i) an indirect ‘virome-bacteriome-metabolite-Treg axis’, and (ii) a direct ‘viral pathogen-associated molecular patterns (PAMPs)-pattern recognition receptors (PRRs)-Treg’ signaling axis. This review comprehensively examines the dualistic role of the gut virome in preserving intestinal equilibrium and its involvement in the pathogenesis or amelioration of intestinal inflammatory disorders such as inflammatory bowel disease (IBD). Furthermore, the influence of the gut virome extends beyond the gut, potentially impacting systemic immune-related diseases. By integrating recent advances in metagenomics, viromics, and immunology, we elucidate the molecular mechanisms through which the gut virome orchestrates immune regulation. This synthesis aims to provide a comprehensive understanding of the gut virome as a critical immune regulator and to explore its potential as a biomarker for disease diagnosis and a novel target for therapeutic intervention.

## INTRODUCTION

The human gastrointestinal tract harbors a vast and diverse community of microorganisms, collectively known as the gut microbiota, which plays a pivotal role in maintaining host health and modulating disease processes. While extensive research has focused on the bacterial component of the gut microbiome, recent advances have highlighted the significance of the gut virome-comprising viruses that infect both bacteria (bacteriophages) and eukaryotic cells-as an integral and dynamic part of this ecosystem^[1,2]^. The gut virome is predominantly composed of bacteriophages, which are highly abundant and exhibit remarkable individual specificity and temporal dynamics^[3,4]^. These viruses are not merely passive inhabitants but actively interact with bacterial hosts and the mammalian immune system, influencing microbial community structure, metabolic functions, and immune homeostasis^[5,6]^. Despite their abundance and potential impact, the gut virome has long been underexplored due to technical challenges in viral detection and characterization, as well as the complexity of virus-host interactions^[7,8]^.

Emerging evidence underscores the gut virome’s critical role in shaping local and systemic immune responses, particularly through its interactions with regulatory T cells (Tregs), a specialized subset of Cluster of Differentiation 4 (CD4)+ T cells essential for maintaining immune tolerance and preventing excessive inflammation^[9,10]^. Tregs are abundant in the intestinal mucosa, where they regulate immune responses to commensal microbes and dietary antigens, thereby preserving gut homeostasis^[11,12]^. Dysregulation of Treg abundance or function has been implicated in the pathogenesis of various inflammatory and autoimmune diseases, including inflammatory bowel disease (IBD), rheumatoid arthritis (RA), and systemic lupus erythematosus (SLE)^[13-15]^. Notably, recent studies suggest that the gut virome can modulate Treg differentiation, recruitment, and suppressive capacity either directly or indirectly via alterations in the bacterial microbiota and microbial metabolites^[16,17]^. For instance, bacteriophages influence bacterial populations that produce short-chain fatty acids (SCFAs), key metabolites known to promote Treg induction and function^[18,19]^. Moreover, specific viral taxa and their encoded auxiliary metabolic genes have been linked to immune modulation and disease states, highlighting the functional relevance of the virome beyond mere taxonomic composition^[20,21]^.

The interplay between the gut virome and Tregs is particularly pertinent in the context of intestinal inflammation and systemic immune-mediated diseases. Alterations in the gut virome composition and diversity have been documented in IBD, with shifts toward increased abundance of Caudovirales phages and decreased Microviridae, correlating with disease activity and mucosal immune responses^[13,22]^. Similarly, in autoimmune conditions such as RA and SLE, gut virome dysbiosis is characterized by depletion of beneficial phages like crAss-like viruses and enrichment of phages targeting pro-inflammatory bacterial taxa, potentially contributing to immune dysregulation^[15,23]^. These virome changes may influence Treg homeostasis and function, thereby exacerbating or ameliorating disease progression^[24]^. Furthermore, the gut virome’’s impact extends beyond the intestine, affecting systemic immunity and distant organ inflammation through modulation of Treg-mediated immune tolerance^[10,25]^.

Technological advancements in metagenomic sequencing and bioinformatics have facilitated the construction of comprehensive gut virome catalogs and enabled longitudinal analyses of virome dynamics in health and disease^[17,26]^. These tools have revealed the high interindividual variability of the gut virome and its sensitivity to environmental factors such as diet, age, geography, and medication, which in turn influence Treg populations and immune regulation^[27,28]^. Despite these advances, challenges remain in standardizing virome sampling and analysis methods, as well as in elucidating the mechanistic pathways linking virome alterations to Treg modulation and disease outcomes^[7,29]^. Addressing these gaps is crucial for harnessing the therapeutic potential of the gut virome, including phage therapy and fecal virome transplantation, aimed at restoring immune balance and treating inflammatory and autoimmune diseases^[30,31]^.

Distinct from previous reviews, this manuscript explicitly separates established mechanisms from testable hypotheses by framing the discussion around two axes: (i) the indirect ‘virome-bacteriome-metabolites-Treg’ axis, which is well-supported by experimental evidence, and (ii) the direct ‘viral PAMPs-PRRs-Treg’ axis, which represents a key frontier for future investigation.

Note on evidence levels: This review distinguishes established mechanisms, inferred mechanisms, and hypothetical mechanisms. Established mechanisms are supported by direct experimental evidence in mammals/humans. Inferred mechanisms are supported by indirect evidence (e.g., correlative human studies). Hypothetical mechanisms lack direct or strong indirect evidence. Readers should interpret all mechanistic statements accordingly.

## COMPOSITION AND ECOLOGICAL CHARACTERISTICS OF THE GUT VIROME

### Major members of the gut virome: bacteriophages and eukaryotic viruses

The gut virome is predominantly composed of bacteriophages (phages) and eukaryotic viruses, each playing distinct roles in host health and disease^[32]^. Bacteriophages constitute the overwhelming majority of the gut virome, often accounting for over 90% of viral particles detected in the intestinal environment^[21]^. Among these, members of the order Caudovirales (tailed phages) are particularly prevalent and exhibit high host specificity, targeting specific bacterial taxa within the gut microbiota^[33]^. This specificity allows phages to modulate bacterial populations and influence microbial community structure, thereby indirectly affecting host immune responses and metabolic functions. For instance, studies have identified dominant phage families, such as Microviridae and Caudovirales, in both human and animal gut samples, with their diversity and abundance fluctuating in response to factors like infection or inflammation^[34,35]^. The high prevalence and diversity of bacteriophages underscore their critical role as regulators of bacterial ecology and modulators of host immunity.

In contrast, eukaryotic viruses, though less abundant, exert significant functional impacts, particularly through direct interactions with host cells. These viruses include enteric pathogens such as noroviruses, rotaviruses, enteroviruses, and astroviruses, which infect intestinal epithelial cells and are implicated in acute gastroenteritis and other diseases^[36-39]^. Notably, some eukaryotic viruses may establish chronic or persistent infections, potentially existing in a symbiotic or commensal relationship with the host. For example, chronic viral infections have been linked to modulation of immune responses and may influence susceptibility to other diseases, including IBD and vaccine responsiveness^[40,41]^. The presence of these viruses in the gut is influenced by environmental exposures, host immunity, and microbial interactions, contributing to the complexity of the gut ecosystem.

A striking feature of the gut virome is its pronounced inter-individual variability, often described as a personalized “viral fingerprint”. This individuality arises from multiple factors including age, diet, geographic location, and host genetic background^[42]^. For instance, infants exhibit a dynamic virome development characterized by increasing richness and abundance of eukaryotic viruses with age, which differs markedly from adult viromes and varies across populations^[43]^. Dietary habits and environmental exposures further shape virome composition, as evidenced by differences in viral communities detected in wastewater, agricultural settings, and urban environments^[44,45]^. Moreover, host genetics influence immune recognition and control of viral populations, thereby affecting virome stability and diversity. This personalized virome landscape has implications for disease susceptibility, immune modulation, and therapeutic interventions.

The dominance of bacteriophages in the gut virome is also reflected in their utility as indicators of fecal contamination and viral pollution in environmental and clinical settings^[46]^. Phages such as crAssphage and pepper mild mottle virus (PMMoV) have been widely studied as molecular markers for human-derived contamination due to their high prevalence and stability in wastewater and environmental waters^[47,48]^. These phages not only serve as proxies for enteric virus presence but also provide insights into viral persistence and removal efficacy in water treatment processes. However, the correlation between phage abundance and eukaryotic viral pathogens is complex and sometimes inconsistent, highlighting the need for comprehensive virome analyses to accurately assess viral risks [Table 1]^[44]^.

**Table 1:** Selected publicly available resources for gut virome analysis

**Resource name**	**Type**	**Key feature**	**Reference**
Gut Virome Database (GVD)	Viral genome catalog	> 80,000 viral genomes from human gut	[42]
Chinese Gut Virus Catalog (CGVC)	Population-specific catalog	16,000+ viral OTUs from Chinese cohorts	[26]
Jovian	Standardized workflow	Reproducible viral metagenomic analysis	[144]
PhRACS	Experimental method	Isolation of phage-host pairs from complex samples	[143]
IMG/VR	Viral genome database	Integrated viral and metagenomic datasets	[163]
Virus-Host DB	Host prediction database	Sequence-based virus-host pairing	[164]

Eukaryotic viruses in the gut virome have been implicated in modulating host immune responses and disease outcomes. For example, enteroviruses such as Coxsackievirus B3 and Enterovirus A71 (EV71) are associated with diseases ranging from hand, foot, and mouth disease to myocarditis and neurological disorders. Recent studies have elucidated mechanisms by which these viruses interact with host cells, including modulation of RNA processing bodies and interference with innate immune signaling pathways, thereby facilitating viral replication and pathogenesis^[49,50]^. Moreover, the presence of certain eukaryotic viruses has been linked to altered vaccine efficacy, as seen in the negative association between enteric virome abundance and oral rotavirus vaccine seroconversion in infants from low- and middle-income countries^[41]^. These findings underscore the functional importance of eukaryotic viruses despite their lower abundance relative to phages.

The gut virome’s composition and dynamics are further influenced by interactions between bacteriophages and eukaryotic viruses, as well as their collective impact on the bacterial microbiome and host immunity. Phages can shape bacterial communities that, in turn, affect viral infection susceptibility and immune responses, while eukaryotic viruses may modulate phage populations indirectly through host immune responses. This intricate interplay contributes to the maintenance of intestinal homeostasis or the development of disease states such as IBD, chronic liver disease, and cancer^[51,52]^. For example, perturbations in the virome, including increased abundance of enteroviruses and altered phage populations, have been observed in IBD patients, suggesting a role for the virome in disease pathogenesis and as a potential therapeutic target^[53]^.

### Ecological dynamics of the virome: lysogenic and lytic cycles

The ecological dynamics of bacteriophages are governed by two principal life cycles: the lytic and lysogenic cycles. In the lytic cycle, phages infect susceptible bacterial hosts, replicate within them, and ultimately cause host cell lysis, releasing progeny virions that can infect new bacterial cells. This direct destruction of bacteria by lytic phages exerts immediate and profound effects on bacterial population structure and community composition. Conversely, the lysogenic cycle involves the integration of the phage genome into the bacterial chromosome, where it exists as a prophage in a dormant state without causing immediate harm to the host. This prophage state allows the phage genome to be vertically transmitted during bacterial replication, effectively becoming a stable genetic element within the bacterial population. The lysogenic cycle thus represents a more covert mode of viral persistence, enabling phages to influence bacterial hosts over extended periods without inducing cell death.

Lysogenic phages play a critical role in horizontal gene transfer among bacteria, mediating the dissemination of genes that can significantly alter bacterial phenotypes and community functions. For example, prophages can carry virulence factors, antibiotic resistance genes, and metabolic traits that enhance bacterial fitness and adaptability. This gene-transfer capacity profoundly impacts bacterial community stability and function, shaping microbial ecosystem dynamics. The integration of prophages into bacterial genomes can also modulate host gene expression and immune evasion strategies, further influencing host-microbe interactions and microbial ecology.

Environmental stressors, such as antibiotic exposure and inflammatory conditions, can trigger prophage induction, shifting lysogenic phages into the lytic cycle. This induction leads to widespread bacterial lysis, causing abrupt changes in bacterial community structure and diversity. Such perturbations can indirectly affect the host immune system by altering the microbial signals that regulate immune responses. For instance, in the gut environment, prophage induction under inflammatory stress may lead to bacterial community dysbiosis, which is closely linked to immune dysregulation and the pathogenesis of inflammatory diseases. The dynamic balance between lysogeny and lysis thus represents a critical regulatory axis in microbial ecology, influencing not only bacterial population dynamics but also host immune homeostasis.

Recent studies have provided empirical evidence supporting these ecological principles. For example, in soil ecosystems, prophage induction has been shown to drive significant viral production and reshape bacterial communities, enhancing nutrient cycling and plant root development^[54]^. This demonstrates how prophage dynamics can influence broader ecosystem functions through microbial community modulation. Similarly, in human-associated microbiomes, the interplay between lysogenic and lytic phages affects bacterial host populations and potentially modulates host health and disease states. The isolation of a novel lytic phage (φPDS1) infecting Parabacteroides distasonis from the human gut highlights the complexity of phage-host interactions and their potential implications for disease-associated bacterial taxa^[55]^.

Moreover, environmental perturbations such as soil wet-up events have been observed to favor lytic viral activity, thereby increasing microbial turnover and nutrient release, underscoring the responsiveness of viral life cycles to external stimuli^[56]^. In the context of viral infections beyond bacteriophages, enteroviruses such as Enterovirus D68 (EV-D68) and EV71 exhibit complex dissemination patterns and interactions with host barriers, although their life cycles differ from bacteriophages^[57,58]^. Nonetheless, these viral dynamics further illustrate the intricate relationships between viruses, microbial communities, and host immune responses.

## INTERACTIONS BETWEEN THE GUT VIROME AND BACTERIOME AND THEIR IMMUNOLOGICAL SIGNIFICANCE

### Bacteriophage-mediated regulation of bacterial communities

Bacteriophages play a pivotal role in shaping the composition, diversity, and abundance of bacterial communities within the gut ecosystem through predator-prey dynamics commonly described as “kill-the-winner” (KTW). This ecological model posits that phages preferentially target the most abundant bacterial populations, thereby preventing any single bacterial species from dominating the community and maintaining microbial diversity and balance. In the gut, this dynamic is critical for sustaining microbial homeostasis and immune equilibrium. Phage predation selectively reduces populations of certain bacterial taxa, including potential pathogens, which helps to preserve the integrity of the gut microbiota and prevent dysbiosis. For instance, phages targeting Proteobacteria, a phylum enriched in some disease states, can modulate their abundance and thus influence host health outcomes^[59]^. The specificity of phage-host interactions allows for precise modulation of bacterial populations, an approach increasingly recognized as a promising therapeutic avenue, especially in conditions such as IBD, where phage therapy may restore microbial balance by targeting pathobionts^[60]^.

Beyond direct bacterial lysis, phages also influence bacterial phenotypes through lysogenic conversion, whereby phage-encoded genes integrate into bacterial genomes and alter bacterial functions. These genes can enhance biofilm formation, modify surface antigenicity, or confer metabolic capabilities, thereby affecting bacterial fitness and interactions with the host immune system. For example, phage-encoded carbohydrate-interacting proteins can mediate phage adherence to the gut mucus layer, indirectly influencing mucosal barrier integrity and immune modulation^[61]^. Such modifications can alter how the host immune system recognizes and responds to bacterial populations, potentially impacting inflammatory processes and disease susceptibility.

The dynamic interplay between phages and bacteria is further complicated by spatial heterogeneity within the gut. Bacteria residing in mucosal biofilms or microcolonies may be shielded from phage predation, creating refuges that allow bacterial persistence despite phage pressure^[62,63]^. This spatial structuring contributes to the coexistence of phages and their bacterial hosts, maintaining microbial diversity and ecosystem stability. Moreover, phage communities themselves are diverse and dynamic, with temperate phages often dominating the gut virome and exhibiting low induction rates, which impose minimal fitness costs on bacterial hosts while enabling genetic exchange and community modulation^[64,65]^.

Environmental factors such as diet, host genetics, and disease states influence phage-bacteria interactions and the resulting microbial community structure. Dietary components, for example, can modulate phage populations and their bacterial hosts. Studies have shown that high-fiber intake increased temperate phage abundance and beneficial bacterial metabolites, correlating with improved host health parameters^[66]^. Similarly, therapeutic interventions like probiotics and fecal microbiota transplantation (FMT) can alter phage-bacteria dynamics, contributing to disease amelioration^[67,68]^. The use of phage cocktails and engineered phages is being explored to selectively target pathogenic bacteria while preserving commensal populations, offering a precision approach to microbiome modulation^[69,70]^.

Overall, bacteriophages regulate gut bacterial communities through direct predation and genetic modulation, maintaining microbial diversity and ecological balance essential for host immune homeostasis. Their ability to selectively reduce pathogenic bacteria and influence bacterial phenotypes makes them critical factors in gut health and promising tools for therapeutic interventions in diseases characterized by microbial dysbiosis. Understanding the complex phage-bacteria-host interactions, including spatial dynamics and environmental influences, is crucial for harnessing phages in clinical applications and advancing microbiome-targeted therapies^[71-73]^.

### Virus-bacteria-host immune triangular dialogue

The intricate interplay among the gut virome, bacterial microbiota, and host immune system constitutes a dynamic triangular dialogue that critically influences intestinal homeostasis and immune regulation, particularly the function of Tregs. Central to this interaction is the bacterial community structure, which directly determines the metabolite profile, including SCFAs and tryptophan derivatives. These metabolites serve as signaling molecules that modulate host immune cells, especially Tregs, thereby maintaining immune tolerance and preventing excessive inflammation. SCFAs (e.g., butyrate, propionate) promote Treg differentiation and function via two distinct mechanisms: (i) histone deacetylase (HDAC) inhibition (epigenetic, nuclear) and (ii) G-protein coupled receptor signaling (e.g., GPR43/FFAR2, GPR41/FFAR3, GPR109A). The relative contribution of each pathway is context- and concentration-dependent, fostering an anti-inflammatory milieu within the gut mucosa. Similarly, tryptophan metabolites, including indole derivatives, engage the aryl hydrocarbon receptor (AhR) on immune cells, further influencing Treg homeostasis and mucosal immunity. The virome, particularly bacteriophages, exerts an indirect but profound influence on this metabolic landscape by reshaping the bacterial community composition. Through selective predation or lysogenic conversion, viruses modulate bacterial abundance and diversity, thereby altering the spectrum and quantity of immunomodulatory metabolites produced. This establishes a cascade of regulation described as the “virome-bacteriome-metabolites-immune cells” axis, underscoring the virome’s role as a master regulator of gut immune equilibrium.

Beyond metabolic modulation, certain bacteriophage particles themselves, or bacterial components released upon phage-induced lysis [such as lipopolysaccharides (LPS) and peptidoglycans], function as pathogen-associated molecular patterns (PAMPs). These PAMPs are recognized by host PRRs, including Toll-like receptors (TLRs) and NOD-like receptors (NLRs), triggering innate immune responses that shape downstream adaptive immunity. This recognition not only initiates protective responses against pathogens but also contributes to immune education and tolerance mechanisms. The complexity of these interactions is exemplified by studies demonstrating that enteric viruses can bind bacterial surface molecules such as lipoteichoic acid, thereby facilitating viral persistence and modulating bacterial colonization, which in turn influences immune activation^[74]^. Moreover, viral infections can disrupt bacterial biofilms, altering microbial community resilience and immune signaling^[75]^. The host’s immune system, in turn, responds to this triad by balancing antiviral defenses with tolerance to commensal bacteria and their metabolites, a process critical for preventing inflammatory diseases such as IBD. Disruption of this delicate balance, whether by viral perturbation of bacterial communities or altered metabolite production, can lead to dysregulated Treg function and chronic intestinal inflammation. Consequently, the virus-bacteria-host immune dialogue represents a sophisticated network of interactions in which the virome indirectly governs immune regulation through bacterial community remodeling and metabolite modulation, while also directly engaging innate immune pathways via PAMP recognition. Understanding this multilayered crosstalk offers promising avenues for therapeutic interventions targeting the gut microbiota and virome to restore immune homeostasis in intestinal and systemic diseases**.**

**Figure 1:**
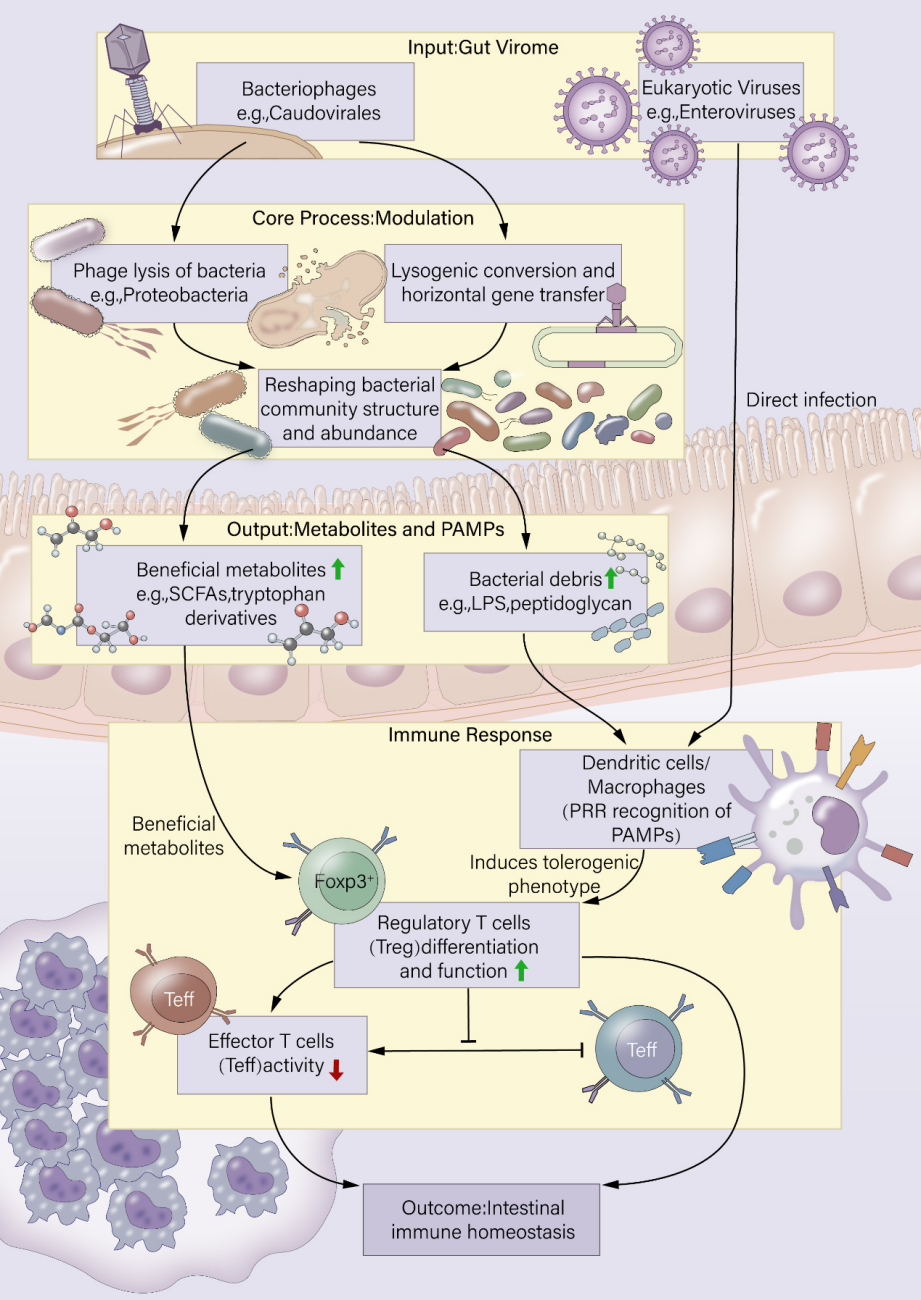
Schematic overview of the triangular crosstalk among the gut virome, bacteriome, and host immune system. SCFA: Short-chain fatty acid; LPS: lipopolysaccharides; PRR: pattern recognition receptor; PAMP: pathogen-associated molecular pattern.

Although this review focuses on the intestinal mucosa, the gut virome may also influence distal mucosal sites via the common mucosal immune system. For example, gut-derived dendritic cells or Tregs expressing gut-homing receptors [C-C chemokine receptor type 9 (CCR9)/α4β7 integrin] can migrate to respiratory or oral mucosa under certain conditions, potentially shaping viral or bacterial tolerance. This concept remains largely theoretical for the virome but is an active area of investigation. [Figure 1]^[74-79]^.

The gut virome, predominantly composed of bacteriophages and eukaryotic viruses, dynamically modulates the bacterial community through predation (“kill-the-winner”) and lysogenic conversion. These interactions reshape bacterial diversity and abundance, thereby altering the production of key immunomodulatory metabolites (e.g., short-chain fatty acids, tryptophan derivatives) and the release of bacterial components (e.g., LPS, peptidoglycan). Metabolites directly promote the differentiation and suppressive function of Tregs, while pathogen-associated molecular patterns (PAMPs) are sensed by PRRs on antigen-presenting cells (APCs), driving a tolerogenic phenotype that further supports Treg induction. Tregs subsequently suppress effector T-cell (Teff) responses, thereby maintaining intestinal immune homeostasis. Eukaryotic viruses may also directly infect host cells and influence local immunity. Solid arrows indicate major pathways; dashed arrows indicate secondary or direct interactions. This integrated axis illustrates how the virome acts as a master regulator of mucosal immune balance. 

## HOST IMMUNE RECOGNITION AND RESPONSE TO THE GUT VIROME

### Innate immune system recognition of viral components

The innate immune system constitutes the first line of defense against viral infections in the gut, where intestinal epithelial cells and underlying innate immune cells express a diverse array of PRRs that detect viral components. Among these PRRs, TLRs such as TLR3, TLR7/8, and TLR9 are pivotal in recognizing viral nucleic acids: TLR3 senses double-stranded RNA (dsRNA), TLR7 and TLR8 detect single-stranded RNA (ssRNA), and TLR9 identifies unmethylated CpG motifs in viral DNA. These receptors are strategically localized in endosomal compartments of intestinal epithelial cells and innate immune cells such as macrophages and dendritic cells, enabling efficient detection of viral genomes and capsid proteins during viral entry or replication^[80-82]^. The recognition of viral nucleic acids by these PRRs initiates signaling cascades that culminate in the activation of transcription factors such as interferon regulatory factors (IRFs) and nuclear factor of kappa light chain enhancer of B cells (NF-κB), leading to the production of type I interferons (IFN-α and IFN-β) and pro-inflammatory cytokines. These cytokines establish an antiviral state by inducing the expression of interferon-stimulated genes (ISGs) that inhibit viral replication and promote the recruitment and activation of additional immune cells, including natural killer (NK) cells and macrophages, thereby shaping the local immune microenvironment^[82,83]^.

Importantly, the innate immune response in the gut must balance effective antiviral defense with tolerance to the abundant commensal virome, including bacteriophages that coexist with the host. Under homeostatic conditions, the intestinal epithelium and mucosal immune system exhibit a degree of immune tolerance toward these symbiotic viral populations. This tolerance is mediated by multiple mechanisms, including the integrity of the epithelial barrier, the protective mucus layer, and the induction of regulatory immune responses that prevent excessive inflammation^[84]^. The mucus layer physically limits viral access to epithelial cells, while epithelial cells themselves can modulate immune responses by producing anti-inflammatory mediators. Moreover, Tregs and tolerogenic dendritic cells help maintain immune homeostasis by suppressing overactive responses to commensal viruses. This immunological equilibrium ensures that antiviral defenses are mounted primarily against pathogenic viruses while avoiding chronic inflammation that could disrupt intestinal homeostasis and contribute to inflammatory diseases^[84]^.

At the molecular level, cytosolic sensors such as retinoic acid-inducible gene I (RIG-I)-like receptors (RLRs), including RIG-I and melanoma differentiation-associated gene 5 (MDA5), complement TLR-mediated sensing by detecting viral RNA in the cytoplasm. These sensors activate downstream signaling pathways involving mitochondrial antiviral signaling protein (MAVS), leading to robust type I interferon responses. The regulation of these pathways is complex; for example, scavenger receptor A (SRA) expressed in macrophages can negatively regulate antiviral signaling by limiting the ubiquitination and activation of TANK-binding kinase 1 (TBK1), thereby modulating IRF3 activation and interferon production^[85]^. Similarly, epigenetic regulators such as KRAB-associated protein 1 (KAP1) suppress the transcription of RIG-I and MDA5, attenuating antiviral responses and facilitating viral immune evasion^[86]^. These regulatory mechanisms highlight the fine-tuning of innate immune sensing to prevent excessive inflammation while maintaining antiviral efficacy.

In addition to nucleic acid sensors, the innate immune system recognizes viral capsid proteins and other structural components. For instance, the Ku heterodimer, traditionally known for its role in DNA repair, functions as a cytosolic DNA sensor that detects viral double-stranded DNA, triggering innate immune signaling. Some viruses, such as vaccinia virus, have evolved proteins (e.g., C4 and C16) that inhibit Ku-mediated DNA sensing to evade immune detection^[87]^. Furthermore, tripartite motif (TRIM) proteins act as multifunctional antiviral effectors by enhancing pro-inflammatory cytokine production, interfering with viral trafficking, and targeting viral proteins for degradation or autophagy, thereby contributing to innate antiviral immunity^[88]^.

The production of type I interferons and pro-inflammatory cytokines following viral recognition not only restricts viral replication but also influences the differentiation and function of adaptive immune cells, including T cells. The local cytokine milieu can direct T cell polarization toward effector or regulatory phenotypes, impacting the balance between immune activation and tolerance in the gut mucosa. For example, type III interferons predominantly act at epithelial barriers to provide localized antiviral protection with limited systemic inflammation, whereas type I interferons can induce broader systemic responses^[89]^. This cytokine-driven shaping of the immune microenvironment is critical in determining the outcome of viral infections and the maintenance of intestinal immune homeostasis.

Moreover, autophagy, an intracellular degradation pathway, intersects with innate immune sensing by delivering viral components to lysosomes for degradation and facilitating the activation of interferon-mediated antiviral responses. Some viruses, such as dengue virus, manipulate autophagy to evade immune detection and promote replication, illustrating the dynamic interplay between viral pathogens and host innate immunity^[90]^. Extracellular vesicles, including exosomes, also participate in innate immune regulation by transporting viral and host molecules that modulate immune cell communication and responses^[91]^.

### Adaptive immunity and the virome

Adaptive immunity plays a critical role in the host defense against enteric viruses, with circulating neutralizing antibodies serving as a key indicator of systemic adaptive immune responses elicited by the virome. Several studies have demonstrated that neutralizing antibodies can specifically target enteroviruses, such as Enterovirus 71 (EV71) and Coxsackievirus A5 (CV-A5), highlighting the virome’s capacity to induce systemic humoral immunity beyond the local intestinal environment. For instance, formalin-inactivated vaccines against EV71 and CV-A5 have been shown to elicit high titers of virus-specific IgG antibodies and neutralizing antibodies in murine models, correlating with protection against lethal viral challenge and reduced viral loads in target organs^[92,93]^. These findings underscore the ability of the virome to prime B cell responses that contribute to systemic immunity, which is essential for controlling viral dissemination and disease progression.

Beyond humoral immunity, the role of T cells, particularly virus-specific T cells, including Tregs, in response to the virome remains less well characterized. Emerging evidence suggests that bacteriophage particles and other viral components can translocate across a compromised intestinal barrier to reach mesenteric lymph nodes and even systemic sites, potentially serving as antigens for T cell recognition. This phenomenon has been observed in models of enterovirus infection where viral replication occurs in multiple tissues, including the small intestine and lymphoid organs, leading to immune activation^[94]^. However, the specificity, phenotype, and functional roles of virome-induced T cells, especially Tregs, which are pivotal in maintaining immune tolerance and preventing excessive inflammation, are still poorly understood. The paucity of data on virome-specific T cell responses represents a significant gap in our understanding of how the adaptive immune system interacts with the intestinal virome.

The virome constitutes a vast reservoir of “non-self” antigens that may influence the pathogenesis of autoimmune diseases through mechanisms such as molecular mimicry and bystander activation. Molecular mimicry involves viral antigens sharing structural similarity with host proteins, potentially triggering autoreactive T-cell responses that contribute to autoimmunity. Bystander activation refers to the nonspecific activation of immune cells in the context of viral infection, which can exacerbate tissue damage and inflammation. Although these mechanisms have been extensively studied in bacterial and viral infections, their relevance to the intestinal virome and their contributions to autoimmune diseases such as IBD and systemic autoimmune disorders remain ripe for exploration. For example, the detection of viral proteins like the hepatitis B virus X protein (HBx) in the gut mucosa of ulcerative colitis patients suggests that viral components within the virome may directly modulate mucosal immunity and inflammation, potentially linking the virome to autoimmune pathogenesis^[95]^. Furthermore, studies on enteric viruses such as murine norovirus have demonstrated that persistent viral infection can induce virus-specific CD8+ T-cell responses that influence intestinal inflammation, implicating the virome in shaping adaptive immune landscapes relevant to autoimmunity^[96]^.

## POTENTIAL MECHANISMS BY WHICH THE GUT VIROME DIRECTLY REGULATES TREGS

### Heterogeneity of tregs in the gut

Tregs are not a uniform population. Thymus-derived Tregs (tTregs, FOXP3+Helios+Neuropilin-1+) dominate systemic immune tolerance, whereas peripherally induced Tregs (pTregs, FOXP3+RORγt+ in the intestine) are highly enriched in the gut mucosa and respond to microbial metabolites such as short-chain fatty acids and retinoic acid. Tissue-resident Tregs further acquire specialized phenotypes (e.g., ST2+ in adipose tissue, GATA3+ in visceral fat). In this review, “Treg” refers to FOXP3+CD4+ cells unless specified; however, where evidence permits, we distinguish tTreg from pTreg and highlight that the gut virome may preferentially affect pTreg induction via bacterial antigen modulation rather than tTreg thymic selection. Mechanistically, virome-induced metabolic shifts, particularly in SCFA levels, are more likely to influence the local proliferation and functional fitness of peripherally induced RORγt+ pTregs within the lamina propria. SCFAs directly promote the *de novo* differentiation of pTregs from conventional CD4+ T cells via histone deacetylase (HDAC) inhibition, a pathway less critical for the maintenance of thymus-derived tTregs. Furthermore, virome dysbiosis may alter the local chemokine milieu (e.g., CCL1, CCL22), thereby affecting the recruitment of circulating tTregs into the gut mucosa. Critically, the balance between Tregs and Th17 cells in the lamina propria is a central determinant of intestinal homeostasis. Virome-driven perturbations - such as the selective depletion of SCFA-producing bacteria - can shift this balance toward a pro-inflammatory Th17-dominant state, which is a well-established driver of IBD pathogenesis discussed in Section **CHANGES AND ROLES OF THE GUT VIROME IN INFLAMMATORY BOWEL DISEASE**.

### Direct effects of virome components on Treg differentiation and function

The direct influence of viral components on Treg differentiation and function represents a critical yet underexplored facet of host immune regulation, particularly within the context of the intestinal virome. Certain eukaryotic viruses, such as herpesviruses, have been documented to encode proteins that mimic host immunoregulatory molecules, including interleukin-10 (IL-10), which is pivotal in promoting immune tolerance and Treg activity. These viral IL-10 homologs can directly modulate immune responses by engaging IL-10 receptors on immune cells, thereby fostering an immunosuppressive environment conducive to viral persistence and potentially enhancing Treg differentiation or function. Moreover, some viruses possess the capacity to infect immune cells directly, inducing a Treg phenotype that may contribute to immune evasion and chronic infection. This phenomenon underscores a sophisticated viral strategy to manipulate host immunity by co-opting regulatory pathways that maintain immune homeostasis.

In contrast, bacteriophages, which do not infect eukaryotic cells, present a more enigmatic role in Treg modulation. Hypothesis (no direct evidence in humans or mice): Phage structural proteins or nucleic acid fragments could theoretically be processed by APCs and presented to T cells either as superantigens or specific antigens, potentially inducing antigen-specific Treg populations. This concept remains speculative and represents a frontier in immunovirology, necessitating rigorous experimental validation to elucidate whether phage-derived components can indeed influence Treg induction and contribute to immune regulation in the gut mucosa.

Furthermore, viral nucleic acids derived from the virome can engage intracellular nucleic acid sensors such as the cyclic guanosine monophosphate–adenosine monophosphate (GMP-AMP) synthase (cGAS)-stimulator of interferon genes (STING) pathway within dendritic cells (DCs). Activation of cGAS-STING signaling by viral DNA or RNA leads to the production of type I interferons and other cytokines that shape DC phenotype and function. This modulation can alter the capacity of DCs to drive Treg differentiation, either promoting or inhibiting the generation of these cells depending on the context and the nature of the viral stimulus. For instance, activation of STING has been shown to enhance antiviral immunity and influence T cell development and effector functions, including those of Tregs, as demonstrated in studies involving EV71 infection models where STING activation suppressed viral replication and modulated immune responses^[97]^. These findings suggest that virome-derived nucleic acids, through intracellular sensing pathways, can indirectly but profoundly impact Treg biology by reprogramming APCs.

Clinical and experimental evidence from enterovirus infections further illustrates the complex interplay between viral components and Treg dynamics. Enterovirus infections in infants have been associated with altered FOXP3 expression in Tregs and shifts in cytokine profiles indicative of disrupted immune regulation, with enterovirus infections correlating with decreased Treg activation and increased proinflammatory T helper 1 (Th1) and T helper 17 (Th17) responses^[98]^. Additionally, EV71 infection has been linked to disturbances in Th cell differentiation and inflammatory cytokine release, implicating viral factors in skewing the balance between effector and Tregs^[99]^. These observations highlight the capacity of viral infections to directly or indirectly modulate Treg function, contributing to immunopathology or immune tolerance depending on the viral and host context.

**Figure 2:**
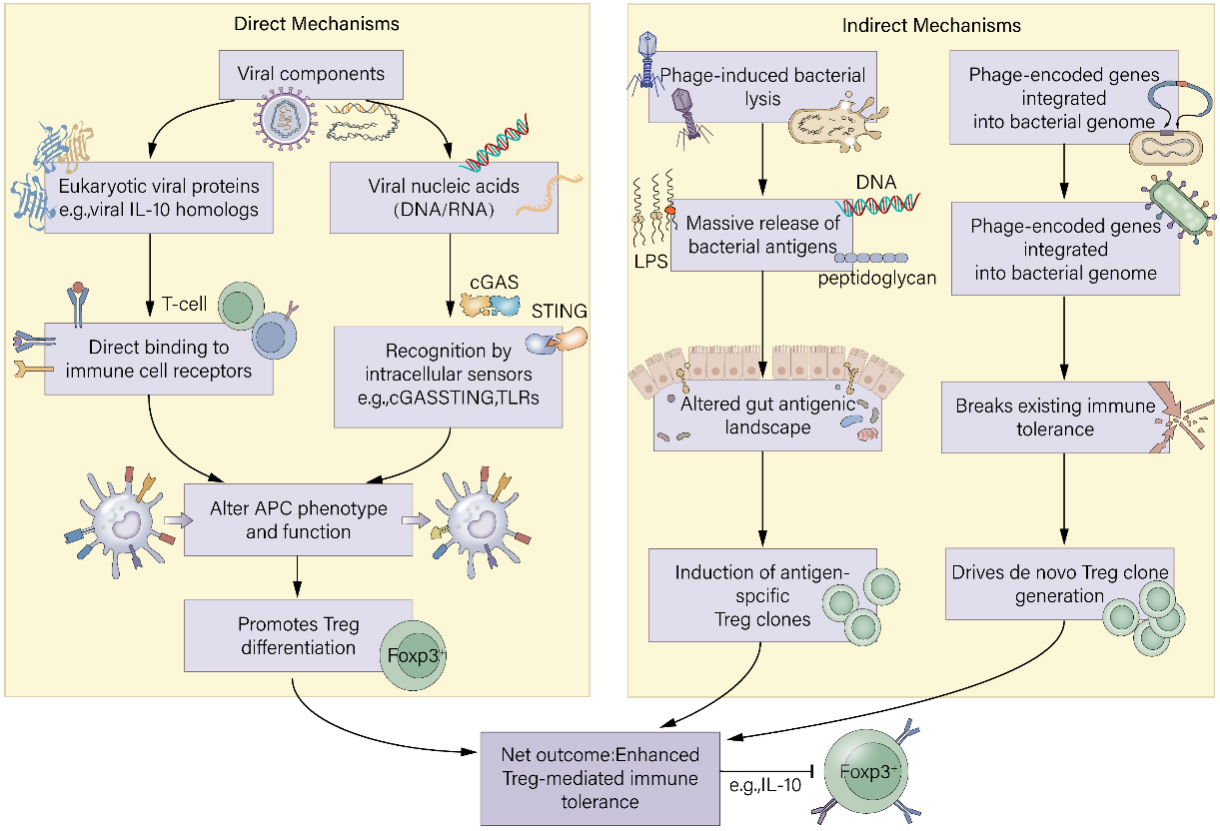
Direct and indirect mechanisms by which the gut virome regulates regulatory T-cell (Treg) dynamics. IL-10: Interleukin-10; cGAS: cyclic GMP-AMP synthase; APC: antigen-presenting cell; STING: stimulator of interferon genes; TLR: toll-like receptor.

Taken together, the direct effects of virome components on Treg differentiation and function encompass viral mimicry of immunoregulatory molecules, potential antigenic stimulation by phage components, and activation of intracellular nucleic acid sensing pathways that reprogram APCs. These mechanisms collectively underscore the virome as a dynamic regulator of Treg-mediated immune homeostasis, with significant implications for intestinal inflammation and systemic immune-mediated diseases. Further mechanistic studies are essential to delineate these pathways and harness this knowledge for therapeutic modulation of Treg responses in virome-associated pathologies [Figure 2].

Direct mechanisms involve viral components that engage host immune cells: eukaryotic viral proteins (e.g., viral IL-10 homologs) can directly bind immune receptors, while viral nucleic acids are detected by intracellular sensors (cGAS-STING, TLRs) in APCs, altering their phenotype and promoting Treg differentiation. Indirect pathways are mediated through phage-bacteria interactions: (i) phage-induced bacterial lysis releases a large pool of bacterial antigens, reshaping the gut antigenic landscape and driving antigen-specific Treg expansion; (ii) phage-encoded genes integrated into bacterial genomes can generate novel bacterial surface antigens, breaking existing tolerance and inducing *de novo* Treg clones. Together, these direct and indirect mechanisms enhance Treg-mediated immune tolerance, crucial for preventing excessive inflammation. 

### Indirect influence on Treg via modification of bacterial antigens

The interaction between bacteriophages and the gut microbiota represents a critical axis through which the intestinal antigenic landscape is dynamically reshaped, thereby indirectly modulating Treg populations. Bacteriophage-mediated bacterial lysis releases substantial quantities of bacterial antigens into the gut lumen, which can significantly alter the composition and abundance of the local antigen pool. This antigenic shift has profound implications for the maintenance of immune tolerance to commensal bacteria, a process heavily reliant on Treg cells. The continuous exposure of the mucosal immune system to a modified antigenic milieu necessitates adaptive adjustments in Treg-mediated tolerance mechanisms to prevent aberrant inflammatory responses that could precipitate or exacerbate intestinal inflammation.

Phagocytosis of enterovirus-infected cells by DCs exemplifies how viral infections can influence antigen presentation and subsequent immune regulation. For instance, human DCs efficiently engulf enterovirus-infected pancreatic beta cells, triggering innate antiviral responses characterized by the induction of RIG-I-like helicases and type I interferon-stimulated genes (ISGs)^[100]^. This process not only establishes an antiviral state but also modulates the antigenic environment encountered by T cells, including Tregs. Similarly, in the context of EV71 infection, plasmacytoid dendritic cells (pDCs) become activated and secrete interferon-alpha, promoting CD4+ T cell proliferation and differentiation^[101]^. However, persistent viral stimulation can lead to functional defects in pDCs and impaired CD4+ T cell tolerance, underscoring the delicate balance between immune activation and regulation.

Bacteriophage activity can alter bacterial antigen pools, potentially mirroring these viral effects by continuously presenting novel or increased quantities of bacterial antigens to the mucosal immune system. This persistent antigenic flux demands robust Treg responses to maintain homeostasis. Indeed, studies have shown that enterovirus infections can disrupt Treg populations; for example, infants with enterovirus infections exhibit decreased FOXP3 expression in highly activated Tregs, accompanied by a proinflammatory cytokine milieu skewed towards Th1 and Th17 responses^[98]^. Such findings suggest that viral infections, and by extension bacteriophage-induced bacterial lysis, can perturb Treg-mediated tolerance by modifying the antigenic stimuli that drive Treg induction and function.

Moreover, the immune response to EV71 infection highlights the complexity of Treg dynamics in the face of altered antigenic exposure. Severe EV71 disease correlates with a reduction in circulating CD4+CD25+FOXP3^high^ Tregs and an increase in Th17 cells, alongside elevated proinflammatory cytokines such as IL-17 and IL-21^[102]^. This imbalance contributes to immunopathology and underscores the importance of Treg-mediated control in preventing excessive inflammation. The modulation of Treg populations in response to changing antigenic landscapes, whether due to viral infection or bacteriophage-mediated bacterial lysis, is thus a critical determinant of intestinal immune homeostasis.

In addition to direct effects on Treg populations, bacteriophage-induced bacterial lysis may influence the antigenic repertoire by releasing bacterial components that serve as ligands for innate immune receptors, such as TLRs and RIG-I-like receptors. For example, in fulminant type 1 diabetes, enterovirus infection induces robust expression of RIG-I and MDA5 in pancreatic islet cells, coupled with infiltration of dendritic cells and macrophages that phagocytose infected cell debris^[103]^. The resultant innate immune activation shapes the adaptive immune response, including Treg activity, by modulating antigen presentation and cytokine environments. Analogously, bacteriophage-mediated bacterial antigen release in the gut could engage innate immune pathways, influencing Treg induction and function indirectly through altered cytokine milieus and APC activation states.

Furthermore, the JanusMatrix immunoinformatics tool has revealed extensive T cell receptor (TCR) cross-reactivity between enteroviral epitopes and those derived from the human microbiome, suggesting that bacteriophage-driven changes in bacterial antigen expression could impact Treg specificity and repertoire^[104]^. This cross-reactivity may facilitate the induction of Tregs that recognize conserved microbial antigens, thereby reinforcing tolerance to commensal bacteria despite antigenic fluctuations caused by bacteriophage activity. Conversely, disruption of this balance could predispose to immune dysregulation and inflammatory diseases.

#### Phage-encoded genes inducing novel bacterial surface antigens and Treg induction

Beyond the direct lytic effects of bacteriophages on bacterial populations, the genetic repertoire encoded by prophages integrated within bacterial genomes can profoundly alter bacterial antigenicity. Phage-encoded genes may confer novel surface antigens or modify existing bacterial surface structures, thereby reshaping the antigenic landscape presented to the host immune system. This antigenic novelty challenges the host’s established immune tolerance mechanisms, compelling the induction of new Treg clones capable of recognizing these altered or novel epitopes to maintain immune homeostasis.

The concept of antigenic variation driven by phage-encoded genes is supported by observations in viral infections, in which the emergence of new viral antigens necessitates adaptive immune recalibration. For instance, in EV71 infection, the activation of innate immune pathways via STING signaling not only restricts viral replication but also modulates T cell development and effector functions, including those of CD8+ T cells and NK cells^[97]^. This modulation reflects the immune system’s capacity to adapt to novel antigenic stimuli, a principle that extends to the bacterial context where phage-encoded antigenic changes may similarly drive T cell repertoire adjustments.

The induction of new Treg clones in response to phage-modified bacterial antigens is critical for sustaining tolerance to the commensal microbiota. Tregs are known to exhibit TCR cross-reactivity, enabling them to recognize a broad spectrum of related epitopes derived from self, commensal, and pathogenic sources^[104]^. This cross-reactivity helps maintain tolerance despite antigenic variability. However, the expression of novel bacterial surface antigens encoded by prophages may exceed the recognition capacity of pre-existing Treg clones, necessitating the *de novo* induction of Tregs specific for these new epitopes. In the tolerogenic gut environment, CD103+ dendritic cells produce retinoic acid and TGF-β, which favor *de novo* Treg induction over effector T cell activation upon encountering novel bacterial antigens. Thus, the outcome is tolerance rather than inflammation.

Experimental models of enterovirus infection provide insights into how immune regulation adapts to novel antigenic challenges. In coxsackievirus B3 (CVB3) infection, the balance between proinflammatory Th1 cells and anti-inflammatory Tregs determines disease outcome. Notably, infection with a CVB3 variant that activates natural killer T (NKT) cells leads to preferential induction of M2 macrophages and CD4+FOXP3+ Tregs, suppressing Th1 responses and myocarditis^[105]^. This cross-regulation highlights the immune system’s plasticity in generating regulatory populations in response to altered antigenic stimuli, a mechanism likely relevant to phage-driven bacterial antigen variation.

Furthermore, adoptive transfer studies demonstrate that Tregs can protect against virus-induced tissue damage by modulating receptor expression and immune responses. For example, Tregs transferred into mice infected with CVB3 reduce viral titers and cardiac inflammation through transforming growth factor-beta (TGF-β)-mediated downregulation of the coxsackie-adenovirus receptor, thereby suppressing immune responses to cardiac tissue while preserving antiviral immunity^[106]^. This immunomodulatory capacity underscores the potential for Tregs to adaptively respond to novel antigens introduced by phage-encoded bacterial modifications, maintaining tissue integrity and immune balance.

In the context of human disease, EV71 infection exemplifies how viral and possibly phage-induced antigenic changes can disrupt Treg homeostasis. Severe EV71 disease is characterized by decreased Treg frequencies and impaired FOXP3 expression, coupled with increased proinflammatory Th17 responses and cytokine storms^[107]^. These alterations suggest that failure to induce or maintain Tregs specific for novel or modified antigens contributes to immunopathology. By analogy, bacteriophage-driven antigenic shifts in the gut microbiota may similarly challenge Treg-mediated tolerance, with implications for intestinal inflammation and systemic immune regulation.

Additionally, the immunomodulatory effects of other immune cells, such as platelets, on T cell differentiation during EV71 infection highlight the complexity of immune regulation in response to antigenic changes. Platelets from infected patients promote Th1 differentiation and interferon-gamma (IFN-γ) secretion, influencing the balance between effector and regulatory responses^[108]^. Such interactions may also be relevant in the gut environment, where phage-induced bacterial antigen variation could modulate multiple immune cell types, collectively shaping Treg induction and function.

## CHANGES AND ROLES OF THE GUT VIROME IN INFLAMMATORY BOWEL DISEASE

### Dysbiotic features of the gut virome in IBD patients

Metagenomic investigations have consistently revealed that patients with IBD [both Crohn’s disease (CD) and ulcerative colitis] exhibit significant alterations in their gut virome, a phenomenon often termed “viral dysbiosis”. This dysbiosis is characterized by increased α-diversity among viral populations and marked shifts in community structure compared to healthy individuals. Notably, the gut virome in IBD is predominantly composed of bacteriophages, especially those belonging to the order Caudovirales, whose relative abundances correlate with disease activity. For instance, studies have reported an expansion of Caudovirales phages in IBD patients, accompanied by a concurrent reduction in Microviridae phages, suggesting a selective enrichment of certain viral taxa linked to disease states^[22,109]^. This viral imbalance is not merely a reflection of bacterial dysbiosis but represents an independent ecological shift, as the virome and bacteriome exhibit distinct dynamics and interactions.

Specifically, Caudovirales phages (tailed bacteriophages) have been implicated in modulating bacterial populations by influencing bacterial diversity, antibiotic resistance, and toxin production, thereby potentially exacerbating intestinal inflammation. Certain phage groups, such as Klebsiella phages, have been associated with disease activity, indicating their possible role as biomarkers or contributors to pathogenesis^[110,111]^. Moreover, eukaryotic viruses, including members of the Herpesviridae family, have been detected at elevated rates in IBD patients’ intestinal mucosa, with Epstein-Barr virus (EBV) and cytomegalovirus (CMV) frequently identified in biopsy samples. These viruses may contribute to mucosal immune dysregulation and inflammation, although their precise pathogenic roles remain to be fully elucidated^[111,112]^.

The dysbiotic virome in IBD is closely intertwined with bacterial community alterations; however, the temporal and compositional changes in the virome do not always parallel those in the bacteriome. For example, in CD, the ileal mucosal virome shows a depletion of both lytic and temperate bacteriophages targeting bacterial pathogens during disease flare-ups, while remission phases exhibit distinct virome-bacteriome ecological patterns, highlighting the complex and dynamic interplay between viruses and bacteria in the gut environment^[110]^. Furthermore, the mucosal virome differs significantly from the fecal virome, with mucosa-associated viruses such as crAss-like phages being abundant yet undetectable in stool samples, underscoring the importance of sampling site in virome studies^[113,114]^.

Beyond the mucosa-versus-fecal distinction, significant spatial heterogeneity exists along the longitudinal axis of the gut. The small intestine (ileum) and colon represent vastly different immunological and microbial niches. The ileum has a thinner mucus layer, higher oxygen tension, and dense lymphoid aggregates (Peyer’s patches), hosting a distinct bacterial community (e.g., enriched in Firmicutes and Proteobacteria) compared to the anaerobic, mucus-thick colon dominated by Bacteroidetes and Clostridia clusters. Consequently, virome-bacteriome-Treg crosstalk is likely site-specific. For instance, the high density of RORγt+ pTregs in the colonic lamina propria may render this site more susceptible to virome-induced shifts in SCFA-producing bacteria, whereas the inductive immune environment of the ileum (dominated by follicular helper T cells and IgA responses) may be more influenced by eukaryotic viruses that directly interact with Peyer’s patches. Future virome studies should therefore carefully account for sampling site, as conflating ileal and colonic signals may obscure disease-relevant associations.

Methodological advances, including signature-protein-based virome profiling, have enhanced the accuracy and efficiency of viral detection and quantification, enabling the identification of disease-specific viral signatures across diverse populations^[115]^. These approaches have revealed consistent viral alterations in IBD cohorts from different geographic regions, reinforcing the notion of a core virome dysbiosis associated with IBD pathogenesis. Importantly, experimental models have demonstrated that transplantation of viromes from IBD patients into germ-free or humanized mice can exacerbate intestinal inflammation, providing causal evidence for the virome’s role in disease progression^[110,116]^.

Where available, evidence is distinguished between CD and ulcerative colitis (UC): CD more consistently shows depletion of Caudovirales targeting Faecalibacterium, whereas UC is associated with expansion of Microviridae. Importantly, mucosal-associated virome (e.g., crAss-like phages) differs significantly from fecal virome, and sampling site (ileal vs. colonic) is a major confounder.

### Hypotheses on mechanisms by which virome dysbiosis influences the occurrence and progression of IBD

#### “Phage-mediated bacterial community disruption” hypothesis

The “phage-mediated bacterial community disruption” hypothesis posits that in IBD, an exacerbation of bacteriophage lytic activity leads to the depletion of protective bacterial populations, such as butyrate-producing Firmicutes, thereby disturbing microbial homeostasis and metabolite balance critical for immune regulation. This disruption undermines the support for Tregs, which is essential for maintaining intestinal immune tolerance, thus exacerbating inflammation. Evidence from CD patients reveals a marked reduction in both lytic and temperate bacteriophages, particularly those targeting pathogenic bacteria, which correlates with disease flare-ups and mucosal inflammation^[110]^. The loss of phages that normally regulate bacterial populations may allow expansion of pathobionts, further destabilizing the microbiota. Moreover, studies in cystic fibrosis children demonstrate decreased abundance of specific bacteriophages such as Faecalibacterium phage FP Taranis, which is linked to reduced beneficial bacterial taxa and altered functional gene profiles related to bacterial cell wall metabolism^[117]^. The depletion of butyrate-producing bacteria, which generate SCFAs critical for Treg differentiation and function, leads to impaired mucosal barrier integrity and heightened immune activation. This is supported by findings that SCFAs and other microbial metabolites have anti-inflammatory effects and promote Treg homeostasis, and their reduction correlates with increased intestinal inflammation^[118]^. Thus, phage-induced bacterial dysbiosis disrupts the metabolic milieu necessary for Treg maintenance, tipping the balance toward effector T cell responses and chronic inflammation characteristic of IBD. The interplay between phages and bacteria is therefore a pivotal factor in microbial ecosystem stability and immune regulation, and its perturbation may be a key driver of IBD pathogenesis.

Extending this hypothesis to clinical therapy, phage-driven depletion of butyrate-producing taxa may directly influence treatment outcomes in IBD. A recent study by Liu *et al.*^[119]^ demonstrates that microbiome-metabolome interactions, particularly those involving SCFAs, regulate the efficacy of infliximab in CD by modulating intestinal immune homeostasis, including Treg function and macrophage polarization. Thus, assessing the abundance of butyrate-targeting phages could serve as a predictive biomarker for anti-TNF responsiveness, and restoring these bacterial populations via phage modulation might represent a novel strategy to overcome non-response to therapy. The disruption of metabolic support not only impairs Treg function but also favors Th17 differentiation, as SCFAs can directly inhibit Th17 polarization. The resultant Treg/Th17 imbalance within the intestinal mucosa is a pivotal event that lowers the threshold for chronic, uncontrolled inflammation characteristic of IBD.

#### “Prophage induction and gene transfer” hypothesis

The “prophage induction and gene transfer” hypothesis suggests that the inflammatory milieu in IBD promotes the induction of temperate phages from lysogenic to lytic cycles, increasing prophage activation and horizontal gene transfer among bacterial populations. This process facilitates the dissemination of virulence factors and antibiotic resistance genes, potentially enhancing bacterial pathogenicity and sustaining aberrant immune activation. In CD, the mucosal virome shows reduced phage diversity but an increase in phages infecting inflammation-associated bacteria, indicating selective pressures favoring prophage induction in pathogenic strains^[120]^. Additionally, gut virome dysbiosis in premature ovarian insufficiency patients shows increased horizontal gene transfer of resistance and virulence genes, highlighting the broader relevance of prophage-mediated gene exchange in disease states^[121]^. The inflammatory environment, characterized by oxidative stress and immune mediators, can trigger prophage induction, leading to bacterial lysis and release of phage particles that infect neighboring bacteria, spreading genetic elements that enhance bacterial fitness and virulence. This dynamic may perpetuate a cycle of microbial imbalance and immune stimulation, as pathogenic bacteria expressing newly acquired virulence traits provoke sustained mucosal immune responses. Furthermore, the increased presence of prophages carrying immunomodulatory genes may directly influence host immune pathways, exacerbating inflammation. The gene transfer facilitated by prophages thus represents a mechanism by which the virome shapes bacterial community structure and function, contributing to the chronicity and severity of IBD by maintaining a pro-inflammatory microbiota and disrupting immune homeostasis.

#### “Immune activation” hypothesis

The “immune activation” hypothesis posits that intestinal barrier disruption in IBD allows increased translocation of viral particles and components from the virome into the mucosa and systemic circulation, leading to excessive activation of innate and adaptive immune responses. This heightened immune stimulation breaks the existing immune tolerance, suppresses Treg function, and promotes effector T cell responses, thereby exacerbating intestinal inflammation. Studies have demonstrated that the IBD mucosal virome is perturbed, with enrichment of enterovirus B species and other eukaryotic viruses that can directly stimulate PRRs such as TLRs and cytosolic sensors, triggering pro-inflammatory signaling cascades^[53]^. Conditional deletion of the vitamin D receptor (VDR) in intestinal epithelial and immune cells alters the virome composition and upregulates viral sensors including TLR3, TLR7, and nucleotide-binding oligomerization domain-containing protein 2 (NOD2), which are implicated in viral recognition and immune activation, further supporting the role of viral components in modulating mucosal immunity^[122]^. The increased viral antigen load due to barrier dysfunction can lead to chronic activation of dendritic cells and macrophages, skewing cytokine profiles toward Th1 and Th17 responses while impairing Treg-mediated suppression. This immune dysregulation is compounded by viral-induced modulation of bacterial communities, which can amplify inflammatory signals. Moreover, in humanized mouse models, IBD-associated viromes provoke inflammation through nucleic acid sensing pathways, and genetic defects in viral sensors such as MDA5 exacerbate epithelial cell dysfunction upon viral exposure^[53]^. Collectively, these findings underscore that virome alterations and enhanced immune recognition of viral components contribute to the breakdown of intestinal immune tolerance, the suppression of Treg activity, and the promotion of pathogenic effector T cell responses, driving the chronic inflammation characteristic of IBD.

**Figure 3:**
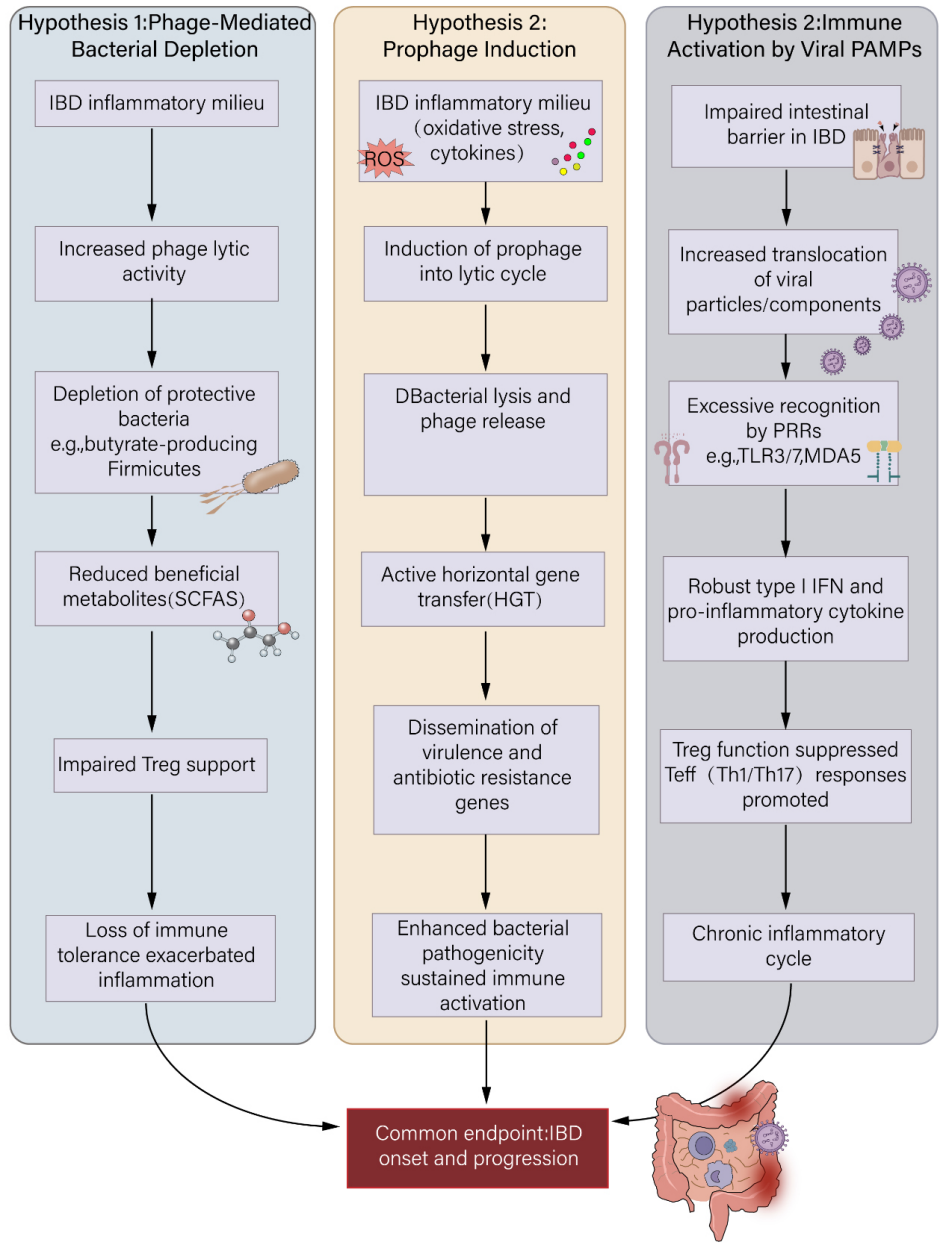
Three non-mutually exclusive hypotheses linking gut virome dysbiosis to IBD pathogenesis. IBD: Inflammatory bowel disease; SCFA: Short-chain fatty acid; ROS: reactive oxygen species; PRR: pattern recognition receptor; TLR: toll-like receptor; MDA5: melanoma differentiation-associated gene 5; IFN: interferon; Treg: T-cell; Teff: effector T-cell; Th1: T helper 1; Th17: T helper 17.

Taken together, these three hypotheses illustrate how virome dysbiosis in IBD can disrupt the delicate balance of the gut ecosystem and immune regulation. Phage-mediated bacterial community disruption diminishes protective bacterial populations and their metabolites essential for Treg support. Prophage induction under inflammatory conditions facilitates horizontal gene transfer, enhancing bacterial pathogenicity and sustaining immune activation. Finally, increased viral translocation and immune recognition due to barrier dysfunction provoke excessive innate and adaptive immune responses, impairing Treg function and promoting inflammation. Understanding these mechanisms provides critical insights into the multifaceted role of the virome in IBD pathogenesis and highlights potential therapeutic targets to restore virome-bacteriome balance and immune homeostasis [Figure 3].

(1) Phage-mediated bacterial community disruption: Enhanced lytic phage activity depletes protective bacteria (e.g., butyrate-producing Firmicutes), reducing beneficial metabolites (SCFAs) and undermining Treg support, thereby exacerbating inflammation; (2) Prophage induction and gene transfer: The inflammatory milieu triggers prophage activation, leading to bacterial lysis and horizontal gene transfer of virulence and antibiotic-resistance genes, which enhances bacterial pathogenicity and sustains immune activation; (3) Immune activation by viral components: Increased intestinal permeability allows translocation of viral particles/components, which are recognized by PRRs (e.g., TLR3/7, MDA5), triggering robust type I interferon and pro-inflammatory cytokine responses that suppress Treg function and promote pathogenic Th1/Th17 responses. These interconnected mechanisms collectively drive chronic intestinal inflammation in IBD.

## THE EXTENDED ROLE OF THE GUT VIROME IN EXTRAINTESTINAL IMMUNE-RELATED DISEASES

### Autoimmune diseases

Emerging evidence increasingly implicates alterations in the gut virome, particularly enteroviruses, as significant contributors to the pathogenesis of various autoimmune diseases such as systemic lupus erythematosus (SLE), rheumatoid arthritis (RA), type 1 diabetes (T1D), and autoimmune thyroid disorders. Studies have demonstrated that patients with these conditions exhibit distinct changes in their gut viral communities, with certain bacteriophage sequences showing cross-reactivity with autoantibodies, suggesting a potential role in initiating or exacerbating autoimmunity. For instance, enterovirus strains isolated from thyroid tissues of patients with autoimmune thyroid diseases (AITD) like Hashimoto’s thyroiditis and Graves’ disease have been shown to persistently infect epithelial cells, inducing transcriptional changes that suppress type I interferon and cytokine pathways while upregulating key immune signaling molecules such as NFKB1/RELA and JAK1/STAT1, which are critical in aberrant T cell activation and inflammatory responses^[123]^. This persistent viral presence may contribute to local tissue damage and the breakdown of immune tolerance, fostering autoimmunity.

In T1D, a prototypical organ-specific autoimmune disease, enteroviruses have been extensively studied as environmental triggers. Detection of enteroviral RNA in peripheral blood mononuclear cells correlates with disease stage and genetic predisposition, particularly variants in the IFIH1 gene, which encodes the viral sensor MDA5, that modulates innate antiviral responses^[124]^. The presence of enteroviral capsid protein VP1 in pancreatic islets from donors with T1D and autoantibody-positive individuals further supports a role for enterovirus in disease progression^[125]^. These infections may induce beta-cell stress and death either directly or via immune-mediated mechanisms, including molecular mimicry, where viral antigens resemble self-proteins, leading to cross-reactive immune responses. Moreover, enterovirus infection can disrupt intestinal barrier integrity, increasing gut permeability and facilitating translocation of microbial products that activate systemic immunity and impair Treg function, thereby compromising peripheral tolerance^[126]^.

The concept of organ-specific axes such as the gut-joint and gut-skin axes highlights the gut virome as a potential mediator of systemic autoimmune manifestations. In diseases like RA and psoriasis, gut microbial dysbiosis is well recognized, but the contribution of the virome remains underexplored. Viral components may serve as unrecognized antigens or signaling molecules that propagate inflammation from the gut to distant sites. For example, in RA, enteroviral infections have been linked to panniculitis and systemic inflammatory symptoms, particularly in immunosuppressed patients, indicating that viral persistence can exacerbate autoimmune pathology^[127]^. Similarly, in dermatomyositis associated with anti-MDA5 autoantibodies, elevated antibody responses to enterovirus B capsid protein VP1 suggest a viral trigger that may initiate or perpetuate autoimmunity^[128]^.

Molecular mimicry is a central mechanism by which the virome may induce autoimmunity. Viral epitopes sharing sequence or structural homology with host proteins can activate autoreactive T and B cells. For instance, cross-reactive epitopes between enteroviruses and human glutamic acid decarboxylase (GAD) have been identified in latent autoimmune diabetes in adults (LADA) and T1D, where antibodies against viral peptides correlate with autoantibody profiles, supporting a viral contribution to disease stratification and progression^[129]^. Additionally, homology between viral proteins and autoantigens such as thyroid-stimulating hormone receptor (TSHR) and insulin-like growth factor 1 receptor (IGF-1R) has been implicated in thyroid eye disease, suggesting that viral infections, such as human papillomavirus, may trigger or exacerbate autoimmune responses via molecular mimicry^[130]^.

Beyond molecular mimicry, enteroviruses may disrupt immune homeostasis by altering the gut barrier and microbiota composition. Coxsackievirus B4 infection in non-obese diabetic (NOD) mice induces dysbiosis characterized by loss of beneficial taxa such as Bifidobacteria and Akkermansia, erosion of the mucosal barrier, and bacterial translocation to pancreatic lymph nodes, which collectively enhance autoreactive immune responses and reduce intestinal Tregs producing IL-10^[131]^. This virus-induced dysbiosis exemplifies how viral infections can synergize with genetic susceptibility to breach central and peripheral tolerance, promoting systemic autoimmunity.

Genetic factors modulating innate antiviral immunity also influence susceptibility to virus-associated autoimmunity. Polymorphisms in genes involved in viral sensing and antiviral pathways, such as IFIH1, tyrosine kinase 2 (TYK2), and others, affect the host’s ability to control enteroviral infections and the subsequent immune response, thereby shaping disease risk and progression^[132]^. Dysregulation of interferon regulatory factors (IRFs), which orchestrate antiviral and inflammatory signaling, further contributes to beta-cell vulnerability and immune activation in T1D^[133]^.

Therapeutically, recognizing enteroviruses as contributors to autoimmune diseases opens avenues for antiviral interventions. Agents such as Vemurafenib have demonstrated efficacy in inhibiting the replication of diabetogenic enteroviruses in intestinal epithelial and pancreatic beta cells, suggesting potential for disease-modifying treatments targeting viral triggers^[134]^. However, challenges remain in diagnosing and managing enteroviral infections in immunosuppressed patients, as exemplified by severe disseminated infections that mimic autoimmune syndromes^[135]^.

### Metabolism and liver diseases

The gut virome, particularly bacteriophages, has emerged as a significant yet underappreciated factor influencing host metabolism and liver disease pathogenesis. In metabolic disorders such as obesity, type 2 diabetes mellitus (T2DM), and non-alcoholic fatty liver disease (NAFLD), alterations in the gut viral community have been consistently observed, with specific phage populations correlating with metabolic parameters. These changes in phage abundance and diversity appear to modulate the composition and function of bacterial communities critical to metabolic homeostasis. For example, phages targeting key bacterial taxa such as Akkermansia and Bacteroides - both known for their roles in mucin degradation and short-chain fatty acid production - may indirectly influence host metabolic status and low-grade systemic inflammation, a hallmark of metabolic syndrome and related liver diseases^[51]^. The interplay between phages and bacteria thus represents a dynamic regulatory axis that can affect the balance between pro-inflammatory and regulatory immune responses, including the function of Tregs, which are often impaired in metabolic inflammation.

Mechanistically, phages can shape bacterial colonization patterns and metabolic outputs by lysing specific bacterial populations or by horizontal gene transfer, thereby altering bacterial gene expression and metabolic pathways. Phage-mediated modulation of the microbiota can influence intestinal barrier integrity and systemic endotoxemia, both of which are implicated in the pathogenesis of metabolic liver diseases. Increased intestinal permeability allows microbial products and possibly viral components to translocate to the liver via the portal circulation, where they can be recognized by hepatic immune cells. This recognition can trigger inflammatory cascades contributing to liver injury, fibrosis, and progression to cirrhosis. Indeed, phages have been shown to migrate beyond the gut lumen into peripheral blood and organs, including the liver, where they may directly interact with hepatic immune cells and influence inflammatory responses^[51]^.

In the context of liver diseases, the gut virome’s role extends beyond indirect modulation through bacterial hosts. Viral components translocated from the gut can be sensed by liver-resident immune cells such as Kupffer cells, dendritic cells, and hepatic stellate cells, potentially exacerbating hepatic inflammation and fibrogenesis. For instance, enteroviruses like coxsackievirus B (CVB) have been implicated in acute and fulminant hepatitis, particularly in neonates, highlighting the direct pathogenic potential of enteric viruses in liver disease^[136]^. Hepatocytes themselves have been shown to trap and silence certain enteroviruses, such as type B coxsackieviruses, acting as a frontline innate immune barrier that protects against systemic viral dissemination but at the cost of hepatocyte injury^[137]^. This antiviral activity of hepatocytes underscores their integral role in innate immunity and suggests that viral-host interactions within the liver are complex and multifaceted.

Furthermore, the integrity of vascular endothelial cells in the liver is critical for controlling viral penetration and subsequent liver pathology. The coxsackievirus and adenovirus receptor (CAR), expressed in endothelial junctions, regulates vascular permeability and viral entry into tissues. Endothelial-specific deletion of CAR increases vascular permeability and facilitates viral dissemination, leading to higher viral loads in the liver and other organs, which exacerbates liver inflammation and injury^[138]^. This finding highlights the importance of endothelial barrier function in modulating the impact of enteric viruses on liver disease progression.

Therapeutically, modulation of the gut virome and its interactions with the host immune system offers promising avenues for managing metabolic and liver diseases. Phage therapy targeting pathogenic bacteria has shown potential in improving gut microbiota composition and reducing inflammation in chronic liver disease models^[51]^. Additionally, natural compounds such as those derived from Artemisia capillaris exhibit antiviral, anti-inflammatory, and hepatoprotective effects, which may be partly mediated through modulation of the gut microbiome and virome^[139]^. Melatonin has also demonstrated antiviral, anti-inflammatory, and immunomodulatory effects in coxsackievirus B3-induced hepatitis, reducing viral loads and liver damage while modulating cytokine profiles and immune cell populations^[136]^. These findings suggest that targeting viral components and their interactions with host metabolism and immunity could be a viable strategy to mitigate liver disease progression.

**Figure 4:**
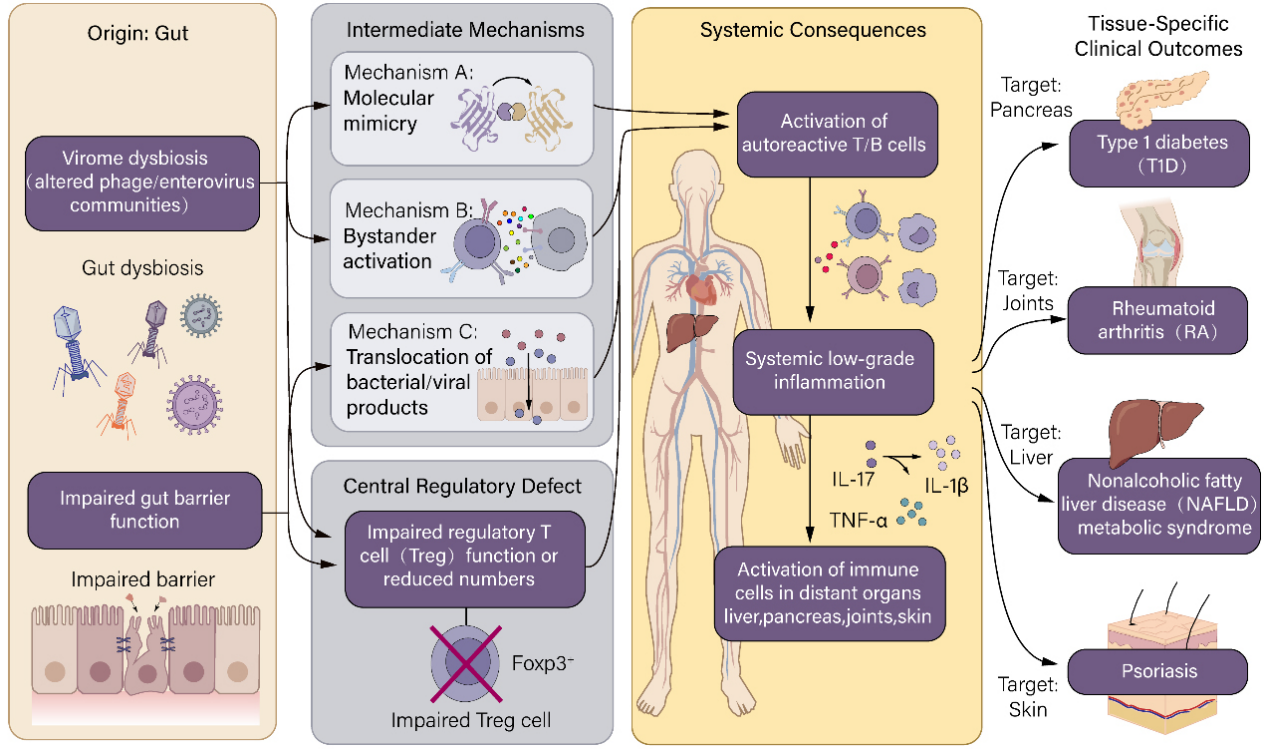
Proposed mechanisms of gut virome involvement in extraintestinal immune-related diseases.

Accordingly, the gut virome, particularly bacteriophages and enteric viruses, plays a crucial role in the pathogenesis of metabolic and liver diseases through complex interactions with bacterial communities, host metabolism, and immune regulation. Alterations in phage populations correlate with metabolic dysfunction and low-grade inflammation, which are closely linked to impaired Treg function. Translocation of viral components to the liver can directly activate hepatic immune cells, contributing to inflammation and fibrosis. Understanding these multifaceted interactions provides new insights into the mechanisms underlying metabolic liver diseases and opens novel therapeutic opportunities targeting the gut virome and its crosstalk with host immunity [Figure 4].

Gut virome dysbiosis, coupled with impaired intestinal barrier function, can drive systemic immune dysregulation through multiple pathways: molecular mimicry, bystander activation, and translocation of bacterial/viral products. These events lead to activation of autoreactive T/B cells, systemic low-grade inflammation, and immune cell activation in distant organs. Consequently, the gut virome has been implicated in the pathogenesis of organ-specific autoimmune diseases (e.g., T1D, rheumatoid arthritis) and metabolic-inflammatory conditions (e.g., non-alcoholic fatty liver disease, psoriasis). A central mechanism across these diseases is the impairment of regulatory T-cell (Treg) function or reduction in Treg numbers, which disrupts peripheral tolerance. The figure highlights the concept of gut-organ axes (e.g., gut-pancreas, gut-joint, gut-liver) through which virome-induced signals propagate systemic disease.

### Emerging evidence for a gut-virome-brain-immune axis

Although direct evidence remains limited, the gut virome could influence CNS immune homeostasis through pathways involving the bacteriome and its metabolites. A growing body of work, summarized by Chetty *et al.*^[140]^, highlights how gut microbial metabolites, particularly SCFAs, can impact neuroinflammation, blood-brain barrier (BBB) integrity, microglial activation, and brain cancer progression. In this context, the gut virome emerges as an upstream regulator. Virome-induced shifts in SCFA-producing bacterial populations can alter the systemic SCFA pool, influencing: (i) BBB permeability by modulating tight junction protein expression; (ii) microglial maturation and their transition from a homeostatic to a neuroinflammatory phenotype; and (iii) peripheral immune cell trafficking into the CNS. Furthermore, virome-driven changes in tryptophan metabolites may signal through the aryl hydrocarbon receptor (AhR) on astrocytes and microglia, modulating their inflammatory tone. Concurrently, systemic type I interferon responses triggered by viral PAMPs that translocate from a dysbiotic gut can prime microglia, exacerbating neuroinflammation. While the trafficking of virome-educated Tregs to the meninges remains speculative, these indirect metabolic and inflammatory pathways provide plausible mechanistic links. Testing these hypotheses will require experimental models where the virome is manipulated independently of the bacteriome.

## TECHNICAL CHALLENGES AND CUTTING-EDGE METHODS IN GUT VIROME RESEARCH

### Technical bottlenecks in viromics analysis

The analysis of viral communities, or viromics, faces several significant technical challenges that hinder comprehensive characterization and understanding of the virome, particularly in complex environments such as the human gut or clinical samples. A fundamental bottleneck arises from the absence of conserved marker genes in viruses analogous to the bacterial 16S rRNA gene, which is widely used for bacterial community profiling. This lack of universal viral markers forces viromics studies to rely heavily on metagenomic sequencing coupled with reference database comparisons for viral identification and classification. However, current viral sequence databases remain incomplete and biased, leading to a substantial proportion of viral sequences being annotated as “unknown” or “viral dark matter”^[141]^. This limitation severely restricts the ability to assign taxonomy and infer biological functions for many viral sequences, impeding insights into viral ecology and host interactions.

Another critical technical hurdle is the complexity of viral particle isolation and purification. Viruses exist in various forms within samples, including intracellular viruses, free virions, and virus-like particles (VLPs), each requiring distinct separation strategies. Efficiently distinguishing these viral entities from host and bacterial DNA contamination is challenging, as nucleic acids from host cells and bacteria can dominate sequencing libraries, diluting viral signals and complicating downstream analyses^[7]^. The purification protocols often involve multiple steps, such as filtration, nuclease treatment, and density gradient centrifugation, which can introduce biases by preferentially enriching certain viral types or losing fragile viral particles. Moreover, the presence of viral nucleic acid fragments from degraded viruses further complicates the interpretation of metagenomic data, as it is difficult to discern whether detected viral sequences represent biologically active viruses or residual genetic material.

A particularly daunting challenge in functional viromics is distinguishing infectious, biologically active viruses from non-infectious viral DNA or RNA fragments. Metagenomic sequencing captures all nucleic acids present, including those from defective or inactivated viruses, which do not contribute to viral replication or pathogenesis. This distinction is crucial for understanding the role of viruses in health and disease but remains technically elusive. Current bioinformatic pipelines and experimental approaches lack robust methods to reliably discriminate active viral infections from background viral debris^[142]^. This limitation affects the interpretation of virome data in clinical diagnostics and epidemiological surveillance, where the presence of viral nucleic acids does not necessarily equate to active infection or disease causation.

Recent methodological advances aim to address these bottlenecks. For example, the PhRACS method exploits specific phage receptor binding protein interactions to isolate phage-host pairs directly from complex samples, bridging the gap between in silico viromics and culture-based validation^[143]^. Additionally, the development of standardized, portable, and reproducible bioinformatic workflows like Jovian facilitates consistent viral metagenomic analyses across different laboratories and computational environments, enhancing data comparability and reliability^[144]^. Despite these improvements, the field still requires comprehensive viral reference databases, improved viral particle purification protocols, and innovative strategies to distinguish active viruses from degraded nucleic acids to fully realize the potential of viromics in understanding viral roles in ecosystems and human health.

### From association to causation: new strategies for functional studies

The transition from identifying correlations between bacteriophages and host bacteria to establishing causative functional relationships represents a critical frontier in understanding the role of phages in modulating host immunity and Treg dynamics, particularly in the context of intestinal inflammation and other diseases. Culturomics approaches have emerged as powerful tools to address this challenge by enabling the development of comprehensive collections of host bacteria and their associated phages. These culture libraries provide essential materials for downstream functional assays, allowing researchers to dissect the specific interactions and effects of individual phages on bacterial hosts and, by extension, on host immune responses. The establishment of such culture collections is particularly important given the complexity and diversity of the gut microbiome and virome, which have historically limited functional investigations due to difficulties in cultivating many microbial constituents.

A gold standard model for demonstrating causality in phage-host interactions involves the use of germ-free or antibiotic-treated animals. These models allow for the controlled colonization of simplified or defined bacterial communities, followed by the introduction of specific phages. This stepwise colonization strategy enables precise manipulation of the microbial ecosystem and the direct assessment of phage effects on bacterial populations and host immune parameters. For example, by colonizing germ-free mice with a defined bacterial consortium and subsequently introducing a targeted phage, researchers can observe changes in bacterial abundance, immune cell populations such as Tregs, and inflammatory markers. This approach has been instrumental in elucidating the causal roles of phages in shaping microbial community structure and modulating host immunity, thereby moving beyond mere associations observed in metagenomic studies.

In addition to in vivo models, innovative molecular tools have been developed to directly manipulate phage populations within complex microbial communities. Phage-targeted editing technologies, such as CRISPR-Cas systems adapted for phage genomes, enable precise genetic modifications of phages, allowing functional dissection of phage genes involved in host interactions. Moreover, the application of phage cocktails - combinations of multiple phages with defined host specificities - serves as an intervention strategy to modulate bacterial communities and assess the functional consequences of phage-mediated bacterial targeting. These interventions can be applied in situ within complex microbiomes, providing a means to validate the roles of specific phages in regulating bacterial populations and host immune responses without requiring complete microbial simplification.

## THERAPEUTIC POTENTIAL AND PROSPECTS OF TARGETING THE GUT VIROME

### Phage therapy and immune modulation

Phage therapy, traditionally focused on the precise lysis of pathogenic bacteria, is increasingly recognized for its complex immunomodulatory effects that extend beyond bacterial eradication. The classical approach involves the use of lytic bacteriophages to target and destroy specific bacterial pathogens, thereby reducing the infection burden. However, during this process, the release of bacterial components such as cell wall fragments and endotoxins, along with the phages themselves, can interact with the host immune system, eliciting immune responses that may be either beneficial or detrimental. Recent studies have highlighted that these immunological side effects require careful reassessment and strategic exploitation to optimize therapeutic outcomes. For instance, phages targeting Cutibacterium acnes in acne have demonstrated not only bacterial lysis but also anti-inflammatory effects by modulating pro-inflammatory cytokine production, suggesting that phages can directly influence immune cell behavior and inflammatory pathways^[145]^. This dual action underscores the need to reconsider phage therapy not merely as an antibacterial tool but as a potential immunomodulatory intervention.

Emerging concepts in phage therapy design emphasize engineering phages to be “immune-neutral” or to possess intrinsic anti-inflammatory adjuvant properties. These engineered phages aim to clear pathogenic bacteria while minimizing pro-inflammatory responses that could exacerbate tissue damage or chronic inflammation. Moreover, these phages could actively promote immune tolerance by enhancing Treg populations or by modulating cytokine milieus to favor anti-inflammatory states. This approach represents a paradigm shift from passive bacterial killing to active immune regulation, potentially improving safety profiles and therapeutic efficacy. For example, phage therapy in methicillin-resistant Staphylococcus aureus (MRSA) infections has shown that certain phages can reduce inflammation and accelerate wound healing, effects that are lost upon phage inactivation, indicating that phage particles themselves modulate immune responses^[146]^. Similarly, phages have been reported to induce macrophage polarization towards anti-inflammatory M2 phenotypes, thereby creating an immune environment conducive to tissue repair and homeostasis, as demonstrated in osteomyelitis models where phage-loaded scaffolds facilitated both bacterial clearance and osteogenesis through immunomodulation^[147]^.

Another innovative immunomodulatory strategy uses phages to indirectly enhance Treg function by manipulating the gut microbiota composition, particularly by modulating the abundance of bacteria that produce beneficial metabolites such as SCFAs. SCFAs promote Treg differentiation and function, contributing to immune tolerance and intestinal homeostasis. Phages can be employed to selectively target bacterial populations that compete with or suppress SCFA-producing commensals, thereby restoring a favorable microbial balance and enhancing Treg-mediated immune regulation. This indirect approach leverages the ecological role of phages in shaping microbial communities to achieve immune modulation without direct interaction with host immune cells. For example, phage therapy targeting Salmonella in murine colitis models not only reduced pathogen load but also modulated immune responses by increasing CD4+ T cell levels and decreasing pro-inflammatory cytokines, illustrating the potential of phages to influence host immunity via microbiome alterations^[148]^. Furthermore, advances in synthetic biology and CRISPR-Cas technologies enable the engineering of phages to deliver immunomodulatory payloads or to selectively edit bacterial genes involved in immune evasion, expanding the scope of phage-mediated immune regulation^[149]^.

Despite these promising developments, challenges remain in fully elucidating the mechanisms by which phages interact with the immune system and in translating these findings into clinical practice. The complexity of phage-host-immune interactions, variability in phage pharmacokinetics, potential immunogenicity of phage particles, and the risk of bacterial resistance necessitate comprehensive preclinical and clinical investigations. Moreover, the design of phage therapies must consider the mode of administration, patient physiological status, and phage biological properties to optimize immunomodulatory effects while ensuring safety^[30]^. The integration of artificial intelligence and machine learning into phage selection and engineering holds promise for the development of personalized phage therapies with tailored immunomodulatory profiles^[150]^.

Clinical translation of phage therapy faces major barriers: narrow host range of lytic phages, rapid emergence of phage-resistant bacteria, endotoxin release from bacterial lysis, immune neutralization of phages, and regulatory hurdles for live viral therapeutics. Patient stratification based on baseline virome and Treg status will be essential.

### Virome as biomarkers and vaccine development

The virome has emerged as a promising source of novel biomarkers and a target for vaccine development, particularly in diseases such as IBD and enterovirus-associated conditions. Specific virome characteristics, including the ratio of bacteriophages to bacteria and indices reflecting core viral populations, hold potential as diagnostic and prognostic biomarkers. For instance, studies have demonstrated that perturbations in the intestinal virome, such as an increased presence of enterovirus B species in IBD patients, correlate with disease phenotypes and inflammatory status, suggesting that virome profiling could aid in disease classification and prognosis prediction^[53,114]^. These viral signatures may complement existing bacterial microbiome markers, offering a more comprehensive understanding of host-microbe interactions in disease contexts.

Mapping the immune memory induced by the virome, including antibody and T cell responses, is another frontier with significant implications. Profiling virus-specific immune responses can reveal disease-specific immunological features, as seen in enterovirus infections where circulating microRNAs and cytokine profiles correlate with disease severity and immune activation^[151,152]^. Such immunoprofiling could identify unique viral epitopes that elicit protective or pathogenic immune responses, informing both diagnostic biomarker discovery and vaccine antigen selection. For example, the identification of immunodominant viral proteins such as EV71 VP1 has guided vaccine design efforts, with VP1-based vaccines demonstrating protective efficacy in preclinical models^[153]^.

Understanding how the virome trains the immune system is critical for developing novel mucosal vaccines and immunotolerance-induction therapies. The mucosal immune system’s interaction with resident viruses shapes immune homeostasis and responsiveness. Experimental evidence shows that adjuvants such as zymosan can enhance mucosal vaccine efficacy by promoting innate immune activation and virus-specific IgA production, as demonstrated in intranasal EV71 vaccine models^[154]^. Moreover, the virome’s role in modulating innate immune pathways, including nucleic acid sensing and cytokine production, suggests that harnessing these interactions could optimize vaccine-induced immunity or induce immune tolerance in inflammatory diseases^[155]^. This concept extends to the development of vaccines that not only prevent infection but also modulate immune responses to maintain mucosal homeostasis.

In vaccine development, the virome’s complexity and diversity present both challenges and opportunities. Current licensed vaccines against enteroviruses, such as inactivated EV71 vaccines, have shown high immunogenicity and safety profiles, significantly reducing disease incidence in vaccinated populations^[156,157]^. However, these vaccines often lack cross-protection against other enterovirus serotypes, necessitating multivalent formulations to address the broad spectrum of circulating viruses^[158,159]^. Innovative vaccine platforms, including live attenuated mutants with improved safety profiles and viral vector-driven replicon systems, are under investigation to enhance immunogenicity and scalability while minimizing biosafety risks^[160,161]^. These approaches leverage detailed knowledge of viral genetics and host-virus interactions to design vaccines that elicit robust and durable immune responses.

Furthermore, the virome’s role as a biomarker extends beyond infectious diseases to immune competence assessment, as seen with anelloviruses such as Torquetenovirus (TTV) and related species serving as indicators of immune status in transplant recipients and may predict vaccine responsiveness^[162]^. This highlights the broader applicability of virome analysis in personalized medicine and immunological monitoring.

Ultimately, the virome offers a rich reservoir of biomarkers for disease diagnosis, stratification, and prognosis, as well as a blueprint for next-generation vaccine development. Integrating virome profiling with immunological mapping and leveraging insights into virus-host immune interactions can drive the creation of targeted, effective vaccines and immunotherapies. Continued research into virome dynamics, immune memory, and mucosal immunomodulation will be pivotal in translating these opportunities into clinical practice, ultimately improving outcomes in IBD, enterovirus infections, and beyond.

Beyond phage therapy, emerging strategies include: (i) fecal virome transplantation (FVT) - early case studies show feasibility, but risks of transferring pathogenic viruses or prophages remain unquantified; (ii) synthetic bacterial consortia engineered to carry immunomodulatory prophages; (iii) viral-vector-based immunomodulation (e.g., adeno-associated virus (AAV) delivering IL-2 variants to expand Tregs locally). These approaches are at preclinical stages but could eventually enable virome-informed precision medicine for Treg-driven diseases.

## CONCLUSION

The gut virome represents a vast and dynamic ecological system whose role extends far beyond a mere collection of viral entities; it functions as a pivotal environmental regulator that shapes immune homeostasis, particularly through modulating Treg dynamics. This nuanced interplay is critical for maintaining intestinal equilibrium, and its disruption is increasingly recognized as a contributing factor in the pathogenesis of IBD and other immune-mediated disorders.

The evidence amassed thus far underscores the multifaceted mechanisms by which the gut virome influences host immunity. Direct interactions between viruses and host cells can trigger immune activation or tolerance, while indirect effects mediated through alterations in bacterial communities further complicate this regulatory network. Such complexity necessitates a balanced interpretation of research findings, as the virome’s impact is context-dependent, varying with viral species, host genetics, and environmental factors. For instance, certain bacteriophages may promote beneficial bacterial populations that support immune regulation, whereas others might exacerbate dysbiosis and inflammation. This duality highlights the importance of integrating diverse research perspectives - from virology, immunology, and microbiome science - to construct a comprehensive understanding of virome-host interactions.

Moreover, the influence of the gut virome transcends the intestinal milieu, implicating systemic immune and metabolic pathways. Associations with extraintestinal autoimmune conditions and metabolic syndromes suggest that the viromeBeyond the mucosa’s regulatory reach is extensive, potentially orchestrating immune responses at distal sites through circulating viral components or immune mediators. This systemic dimension adds another layer of complexity, challenging researchers to delineate causal relationships amid a web of interconnected biological processes.

Current research is progressively shifting from descriptive studies that catalog virome composition toward mechanistic investigations that elucidate causal pathways. Advances in high-throughput sequencing, viral metagenomics, and single-cell immunophenotyping are enabling more precise characterization of viral populations and their functional roles. However, significant technical and theoretical challenges remain. The vast diversity and rapid evolution of viruses complicate their identification and functional annotation. Additionally, the lack of standardized methodologies and the difficulty in establishing causality in complex host-microbe interactions hinder the translation of findings into clinical applications.

Looking forward, the therapeutic potential of targeting the gut virome is a promising frontier. Precision phage therapy, engineered viral particles, and virome modulation strategies offer innovative avenues to restore immune balance and treat inflammatory diseases. These approaches could complement or surpass current microbiome-based interventions by directly manipulating viral constituents that influence bacterial communities and immune responses. Furthermore, comprehensive virome atlases hold promise as diagnostic tools, enabling early detection and personalized management of immune-related disorders.

In conclusion, the gut virome emerges as a critical regulator of immune homeostasis with profound implications for health and disease. A balanced synthesis of current research reveals a complex, bidirectional relationship between viruses, the microbiota, and the host immune system that demands interdisciplinary investigation. Overcoming existing methodological hurdles and deepening mechanistic insights will be essential to harness the virome’s full potential in clinical practice. Ultimately, elucidating the virome’s role will not only advance immunology and microbiology but also pave the way for novel diagnostic and therapeutic strategies that address the root causes of immune dysregulation and inflammation.

## DECLARATIONS

### Copyright

© The Author(s) 2026.
